# Characterizing the Anticancer Treatment Trajectory and Pattern in Patients Receiving Chemotherapy for Cancer Using Harmonized Observational Databases: Retrospective Study

**DOI:** 10.2196/25035

**Published:** 2021-04-06

**Authors:** Hokyun Jeon, Seng Chan You, Seok Yun Kang, Seung In Seo, Jeremy L Warner, Rimma Belenkaya, Rae Woong Park

**Affiliations:** 1 Department of Biomedical Sciences Ajou University School of Medicine Suwon, Gyeonggi-do Republic of Korea; 2 Department of Biomedical Informatics Ajou University School of Medicine Suwon, Gyeonggi-do Republic of Korea; 3 Department of Preventive Medicine Yonsei University College of Medicine Seoul Republic of Korea; 4 Department of Hematology-Oncology Ajou University School of Medicine Suwon, Gyeonggi-do Republic of Korea; 5 Department of Internal Medicine Kangdong Sacred Heart Hospital Hallym University College of Medicine Seoul Republic of Korea; 6 Division of Hematology and Oncology Department of Medicine Vanderbilt University Medical Center Nashville, TN United States; 7 Department of Health Informatics Memorial Sloan Kettering Cancer Center New York, NY United States

**Keywords:** antineoplastic combined chemotherapy protocols, electronic health record, cancer, pattern, chemotherapy, database, retrospective, algorithm, scalability, interoperability

## Abstract

**Background:**

Accurate and rapid clinical decisions based on real-world evidence are essential for patients with cancer. However, the complexity of chemotherapy regimens for cancer impedes retrospective research that uses observational health databases.

**Objective:**

The aim of this study is to compare the anticancer treatment trajectories and patterns of clinical events according to regimen type using the chemotherapy episodes determined by an algorithm.

**Methods:**

We developed an algorithm to extract the regimen-level abstracted chemotherapy episodes from medication records in a conventional Observational Medical Outcomes Partnership (OMOP) common data model (CDM) database. The algorithm was validated on the Ajou University School Of Medicine (AUSOM) database by manual review of clinical notes. Using the algorithm, we extracted episodes of chemotherapy from patients in the EHR database and the claims database. We also developed an application software for visualizing the chemotherapy treatment patterns based on the treatment episodes in the OMOP-CDM database. Using this software, we generated the trends in the types of regimen used in the institutions, the patterns of the iterative chemotherapy use, and the trajectories of cancer treatment in two EHR-based OMOP-CDM databases. As a pilot study, the time of onset of chemotherapy-induced neutropenia according to regimen was measured using the AUSOM database. The anticancer treatment trajectories for patients with COVID-19 were also visualized based on the nationwide claims database.

**Results:**

We generated 178,360 treatment episodes for patients with colorectal, breast, and lung cancer for 85 different regimens. The algorithm precisely identified the type of chemotherapy regimen in 400 patients (average positive predictive value >98%). The trends in the use of routine clinical chemotherapy regimens from 2008-2018 were identified for 8236 patients. For a total of 12 regimens (those administered to the largest proportion of patients), the number of repeated treatments was concordant with the protocols for standard chemotherapy regimens for certain cases. In addition, the anticancer treatment trajectories for 8315 patients were shown, including 62 patients with COVID-19. A comparative analysis of neutropenia showed that its onset in colorectal cancer regimens tended to cluster between days 9-15, whereas it tended to cluster between days 2-8 for certain regimens for breast cancer or lung cancer.

**Conclusions:**

We propose a method for generating chemotherapy episodes for introduction into the oncology extension module of the OMOP-CDM databases. These proof-of-concept studies demonstrated the usability, scalability, and interoperability of the proposed framework through a distributed research network.

## Introduction

### Background

In cancer research, real-world data, with the exception of cancer registries, have been relatively underused despite recent advances in information technology and the availability of data from electronic health records (EHRs) or administrative claims databases [1]. One of the major challenges to the active use of EHRs or claims databases in cancer research is the limited availability of clinically relevant structured data elements. The essential data elements for cancer research include records of anticancer treatment at the unit of the regimen, rather than the individual drugs used in the regimen. In order for researchers to obtain the data necessary for conducting a comparative study of the treatment regimens of patients with cancer, a labor-intensive preprocess is inevitable [2-4].

### Prior Work

Previously, researchers developed algorithms to replace the manual endeavor of capturing the details of chemotherapy from medication histories [5-8]. Even though these studies carved paths toward the use of real-world evidence in cancer research, none of them focused on identifying and addressing organizational barriers. Due to heterogeneity in the structure and semantics of EHRs or claims databases across institutions and countries, none of these studies provided a scalable framework.

The Observational Health Data Sciences and Informatics (OHDSI) collaboration, which is a multistakeholder group organized for global collaboration studies, provides the Observational Medical Outcomes Partnership (OMOP) Common Data Model (CDM) to contribute to medical research on harmonized observational databases [9]. The oncology work group in the OHDSI community has proposed an oncology module to facilitate the difficult task of collecting oncology data [10]. The oncology module has a structure for populating chemotherapy episodes and a vocabulary for hematology/oncology [11]. However, a generalizable method of populating chemotherapy episodes has not been proposed, and cases involving the use of data schema are scarce.

### Objectives

The main objective of this study was the seamless introduction of the oncology extension into OMOP-CDM by developing an algorithm to automatically identify regimen-level chemotherapy episodes among patients with cancer. To conduct proof-of-concept studies of the availability of the generated chemotherapy episodes, the treatment patterns and trajectories of patients with cancer are presented by additional software. We also identified differences in the onset time and incidence of neutropenia events in patients according to different routine regimens.

## Methods

### Study Design

This study was composed of two main processes: (1) the development of an algorithm to identify anticancer treatment episodes from the OMOP-CDM database and (2) the analysis of the trends and trajectories in cancer treatment or clinical events based on the algorithm-derived episode records using the visualization software. Furthermore, we performed a pilot study to identify the time of neutropenia onset across various chemotherapy regimens, to validate the scalability of the algorithm. All methods were independently applied to each database and data were collected exclusively as graphical summaries.

### Data Sources

We conducted this study using two EHRs of Korean tertiary hospitals and a nationwide claims database from South Korea. The Ajou University School of Medicine (AUSOM) database includes the medical records of 3.14 million patients collected from 1994-2018 [12]. The Kangdong Sacred Heart Hospital (KDH) database includes the medical records of 1.68 million patients collected from 1986-2018. The Health Insurance Review and Assessment Service (HIRA) COVID-19 data set is a nationwide administrative claims database that provides information on reimbursed insurance claims from 2017-2020 for 7590 patients with COVID-19 in South Korea [13]. Each data source was standardized into the OMOP-CDM database (version 5.3). According to the medical history and diagnosis, we identified patients with lung, breast, and colorectal cancer from the EHR databases. From the HIRA COVID-19 data set, we selected patients with COVID-19 and any malignant neoplasm disease as the targets of the descriptive analysis.

### Algorithm Development

#### Workflow of the Chemotherapy Regimen Extraction

**Figure 1:**
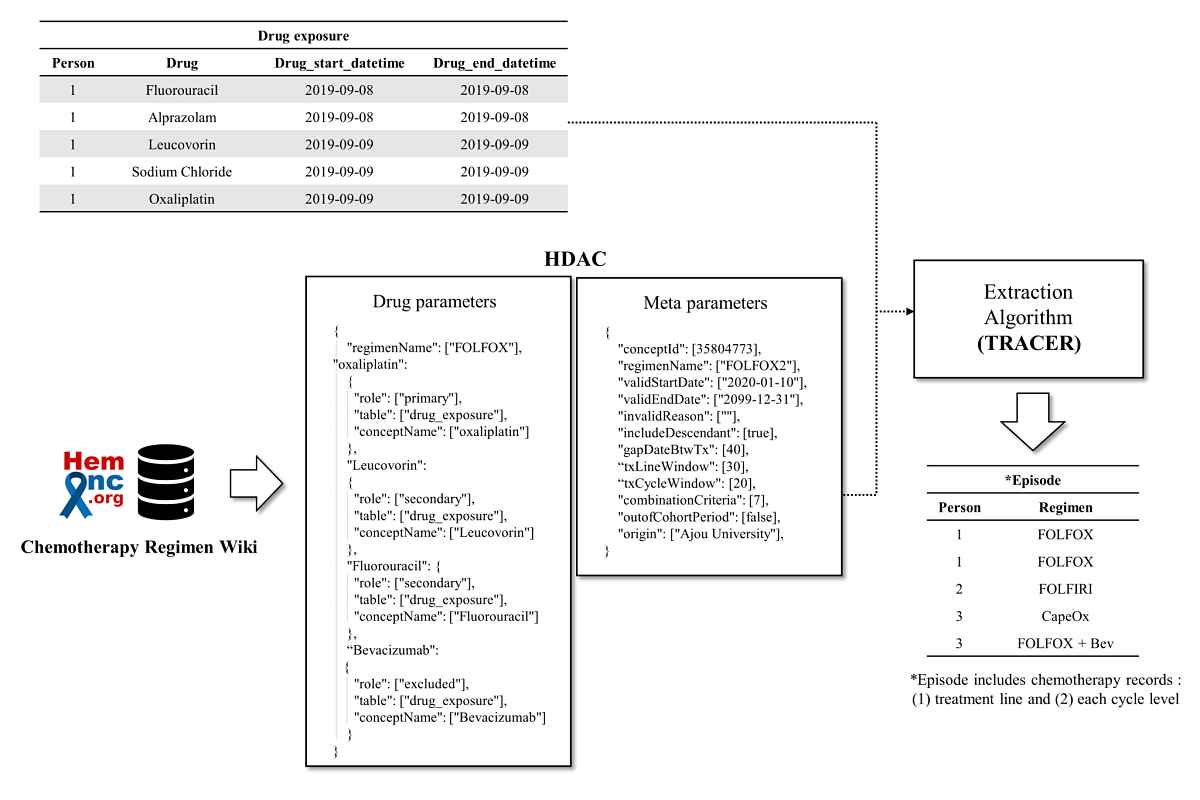
Schematic workflow of the chemotherapy episode extraction. A total of 1506 regimen protocols from HemOnc (a web-based open-source database of cancer chemotherapy regimens) were parameterized as JSON structured data, which is termed HDAC. The JSON file and single drug exposure records in the OMOP-CDM database were instantiated as input data for an algorithm. The TRACER identified chemotherapy episodes by leveraging the parameters from HDAC. The chemotherapy episodes were curated in the episode table, which is an oncology module in the OMOP-CDM. Bev: bevacizumab; CDM: Common Data Model; FOLFIRI: fluorouracil, leucovorin, and irinotecan; FOLFOX: fluorouracil, leucovorin, and oxaliplatin; HDAC: Hierarchical Description for Administration of Chemotherapy; JSON: JavaScript Object Notation; OMOP: Observational Medical Outcomes Partnership; TRACER: Tool for Regimen-level Abstraction of Chemotherapy Episode Record.

Figure 1 shows the entire process used for generating chemotherapy episodes from medication records in harmonized databases. HemOnc is a standard vocabulary adopted by the OMOP-CDM for anticancer agents and chemotherapy regimens derived from the homonymous wiki page, including freely available medical sources for regimens and general information [14]. To convey the semantic regimen protocols in HemOnc to the programmatic algorithm, the section of the protocols detailing the regimen was collected and converted to parameterized values that list the component drugs as well as appropriate time criteria that reflect the drug schedule. Hence, we developed the Hierarchical Description for Administration of Chemotherapy (HDAC), which is a machine-readable JavaScript Object Notation (JSON) snippet containing the parameterized information of HemOnc. The HDAC contains the variables for the constituent drug of the regimen and the time-range value parameters for the temporal window of the medication schedule.

**Figure 2:**
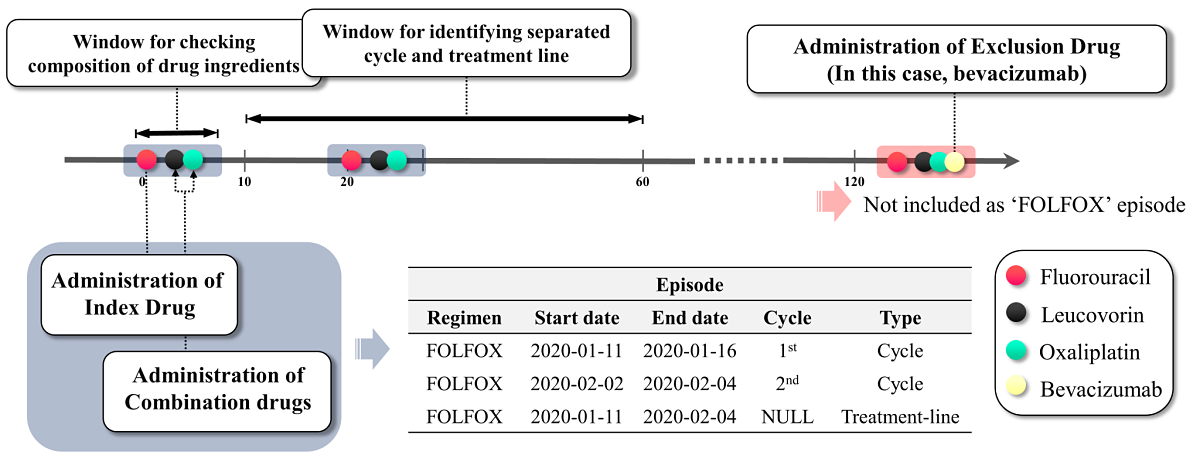
Schematic depiction of algorithm rules in a tool for regimen-level abstraction of chemotherapy episode records. FOLFOX: fluorouracil, leucovorin, and oxaliplatin.

We developed a Tool for Regimen-level Abstraction of Chemotherapy Episode Records (TRACER) to populate the regimen-level abstracted chemotherapy episodes (Figure 2). The TRACER generated the episodes by leveraging the parameter values of drug conditions or temporal window in HDAC. The chemotherapy episodes included the type of regimen and the number of treatment cycles.

#### Instantiation of Chemotherapy Regimen Descriptions

The HDAC includes standardized parameters to feed the specifications of regimen protocols into the algorithm. The variables in HDAC are categorized into two types: parameters for drug composition (drug parameters) and meta parameters.

The drug parameter includes the identifiers of drugs (OMOP concept IDs), which link a specific regimen with its respective role. Each drug parameter is granted a role as an index drug, combination drug, or exclusion drug. The index drug in the HDAC is a constituent drug that can be used to identify the first day (day 1) of treatment. A combination drug in the HDAC is a constituent drug (other than the index drug) of the regimen. An exclusion drug is one whose appearance would indicate another regimen. For example, oxaliplatin is the index drug in the fluorouracil, leucovorin, and oxaliplatin (FOLFOX) regimen. Leucovorin and fluorouracil are combination drugs. In this example, bevacizumab is considered as an exclusion drug to distinguish the FOLFOX regimen from the FOLFOX-bevacizumab regimen (Figure 2).

The meta parameter includes the metadata of the HDAC document (eg, origin, valid date, or invalid reason for document) to determine the modifications and define the window range to be adjusted to the algorithm rule. The window identifies a unit of drug records that determines whether the medication record is a part of a particular regimen or distinguishes a boundary for a distinct treatment cycle. The HDAC also stipulates the window for episodes to distinguish the separated treatment line. The concept ID (encoded in the HemOnc vocabulary) of the drug regimen is a primary key for each HDAC snippet. Based on the chemotherapy indications in the HemOnc web database, a total of 1506 indications for chemotherapy protocols were reviewed by an expert and instantiated into the HDAC.

#### Definitions of the Algorithm

**Figure 3:**
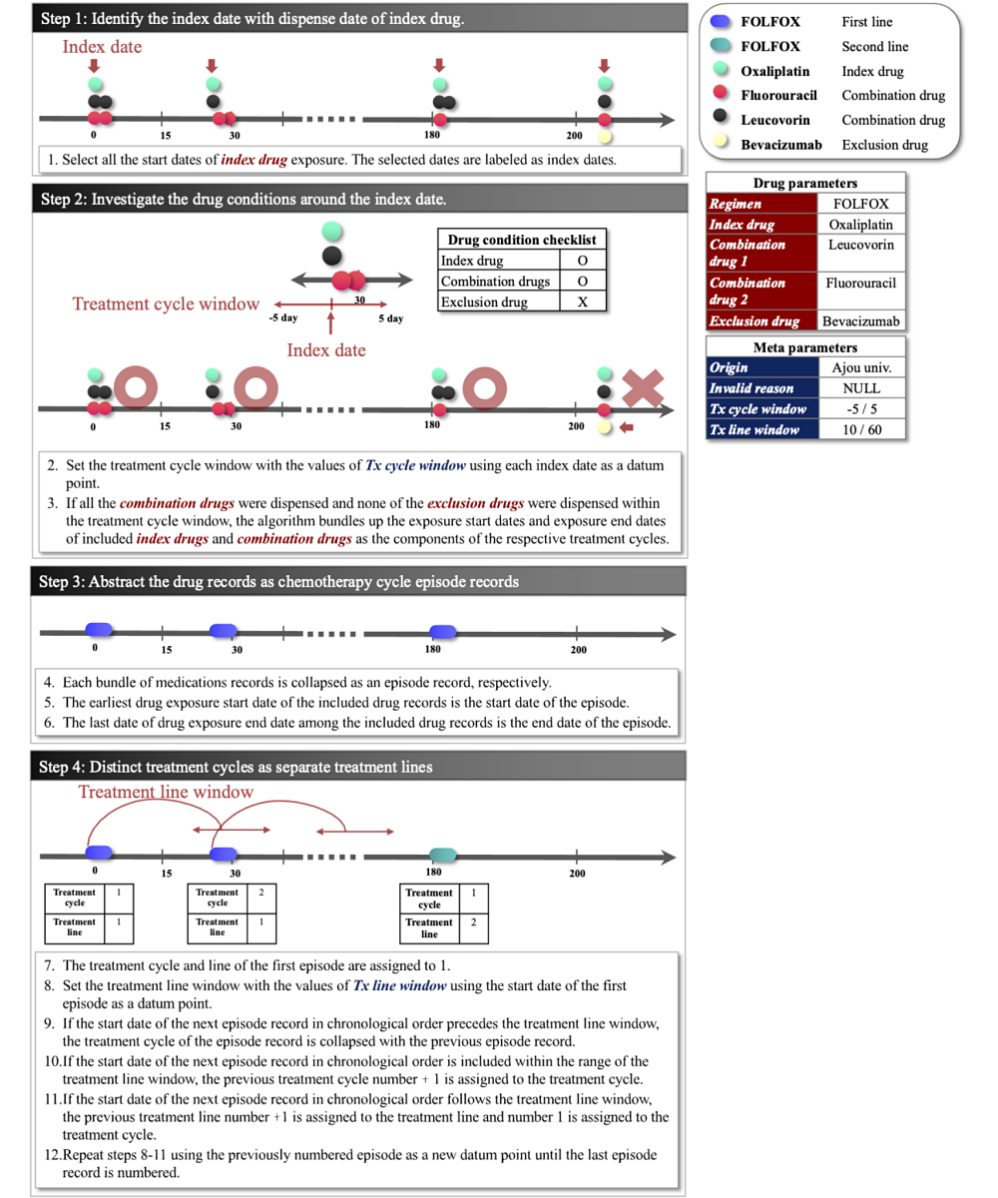
Definition of chemotherapy regimen episode extraction algorithm. FOLFIRI: fluorouracil, leucovorin, and irinotecan; FOLFOX: fluorouracil, leucovorin, and oxaliplatin; HDAC: Hierarchical Description for Administration of Chemotherapy.

The TRACER sequentially extracts the episodes of regimens included in a list of user settings. The algorithm identifies each treatment cycle episode record and treatment line episode of regimens with the defined rules and parameters in a step-by-step manner (Figure 3). The algorithm consists of the following four steps:

1. Day 1 of the respective treatment cycle (index date) is identified based on the dispense date of the index drug. Each index date is flagged as a datum point for checking the use of other drug ingredients to identify a specific regimen.
2. The prescriptions of the combination drug or exclusion drug are investigated within the period of the predefined window for the cycle in HDAC. If the index drug and all combination drugs were given and none of the exclusion drugs were prescribed in this period, the records of the index drug and combination drug are regarded as a constituent component for a targeting regimen. These records are abstracted as a regimen episode record.
3. The start date of each episode is derived from the start date of the instance of index drug utilization, and the end date of the episode record is derived from the end date of the last index or combination drug utilization. The generated episodes are curated in the episode table of the OMOP-CDM oncology module.
4. The episodes are numbered sequentially in chronological order, provided the interval between the start dates of each episode does not exceed the predefined window in the HDAC. The episode records are collapsed as an identical episode when the interval exceeds the cycle window. The window for distinguishing the treatment line is also defined in the HDAC. The TRACER distinguishes the different treatment lines by changes in regimen type or by episodes beyond the window for the treatment line based on the start date of the previous episode.

### Algorithm Validation

We reviewed the discharge and progress notes of patients to validate the accuracy of the proposed algorithm. The following regimens were validated: (1) fluorouracil and folinic acid (FULV), (2) FOLFOX, (3) fluorouracil, leucovorin, and irinotecan (FOLFIRI), and (4) capecitabine monotherapy. Among patients with records of algorithm-derived episodes on target regimens, 100 patients were randomly selected for each type of regimen. For this population, we examined the episode records and compared them to clinical notes.

### Characterizing the Treatment Patterns and Trajectories

We developed visualization applications for characterizing the treatment patterns for patients with cancer treated with chemotherapy. Using the tool, we present the relative proportions of the use of the regimens across all chemotherapy treatments from 2008-2018. The distributions of the iterated number of treatment cycles for each patient according to regimen type were portrayed as a heat map with color saturation varying according to the number of patients. The anticancer treatment trajectories were also illustrated for patients who received routine anticancer treatment according to the type of cancer. The trajectories of patients with COVID-19 and cancer were added, to validate the scalability of the tools. The descriptive results of EHR databases included the eight most prevalent regimen types. For the AUSOM database, we added hormone therapies for breast cancer and targeted therapies or immunotherapy for lung cancer in the descriptive analysis.

### Timing of Neutropenia Onset Analysis

We also conducted a pilot study that investigated the timing of chemotherapy-induced (febrile) neutropenia (CIN/FN) events from the first chemotherapy episode. Neutropenia is a common adverse event of myelosuppressive chemotherapy. In compliance with National Cancer Institute Common Terminology Criteria for Adverse Events (CTCAE; version 5.0) CIN grade 4 (absolute neutrophil count [ANC] <0.5 × 10^9^/L) was used to identify severe CIN events. FN events were identified as ANC of <1.0 × 10^9^/L with a diagnosis of fever or infection, or any use of granulocyte colony-stimulating factor prophylaxis. A plot showing each neutropenia event as a dot by date of onset was displayed, and in the same plot, a violin plot showed the trends in date of neutropenia onset. To identify the onset time of the neutropenic event, the gap between the date of the first chemotherapy treatment and the date of the first CIN/FN onset was calculated for each patient. As the overall measurement schedule was weekly, the onset dates of neutropenia were categorized in 7-day segments to show the trends of onset dates rather than definite dates. To clarify the effect of a single drug regimen on neutropenia onset, the chemotherapies are limited to the first-line treatment and only the CIN/FN events within 30 days of initiation of chemotherapy were considered. On the day of chemotherapy, the ANC level might be temporarily lowered; therefore, a CIN/FN event during chemotherapy administration was ignored. We also depicted the incidence of CIN/FN events in each cycle by regimen type. The four regimens with the highest frequency of CIN/FN events by type of cancer were included for the incidence plot.

### Statistical Analysis

The descriptive analysis was conducted using chemotherapy episodes that were derived from the algorithm. To validate the algorithm, we calculated the proportion of patients who had episodes with identical regimen types as described in clinical notes. The mean absolute error (ie, the mean of the absolute values for the differences between the estimated number of cycles and the actual records in the clinical notes) and the root mean square error (ie, the square root of the mean of the differences between the estimated number of cycles and the actual records in the clinical notes) were also calculated. The overall system was built using R (version 3.5.2; R Foundation for Statistical Computing). The source codes for the algorithm and visualization software have been uploaded to GitHub [15].

### Ethics Statement

This study was approved by the institutional review board of Ajou University Hospital of the Republic of Korea (approval number: AJIRB-MED-OBS-20-092) and Kangdong Sacred Heart Hospital of the Republic of Korea (approval number: 2017-03-003). The institutional review board number for the use of HIRA data was AJIRB-MED-EXP-20-087.

## Results

### Population Characteristics

The TRACER generated a total of 178,360 chemotherapy episodes from the AUSOM database. The episodes consisted of 12 regimen types for colorectal cancer, 24 types for breast cancer (including 6 types of hormone therapy regimen), and 19 types for lung cancer (including 8 types of targeted therapy regimen). The number of patients who were treated with the respective regimens are listed in Multimedia Appendix 1. The characteristics of patients in the AUSOM database are listed (Table 1). Among the 10,353 colorectal, 9546 breast, and 12,671 lung cancer cases, 3151 (30.4%), 5568 (58.3%), and 1593 (12.5%) patients had records of a treatment episode, respectively. The number of patients treated with chemotherapy increased over the years. A total of 69,353 chemotherapy episodes were extracted from the KDH database. The KDH database included 2758 patients with colorectal cancer, 564 (20.4%) of whom had chemotherapy episodes. Among the patients with breast cancer (n=6420) and lung cancer (n=2663) in the KDH database, chemotherapy episodes were identified for 1075 (16.7%) and 642 (24.1%) patients, respectively. The HIRA COVID-19 data set mostly consisted of female patients (60%) [13]. Among 7590 patients with COVID-19, we identified 382 (5%) patients with a primary malignant neoplastic disease.

**Table 1:** Characteristics of patients with colorectal, breast, and lung cancer in the Ajou University School of Medicine database.

Characteristics of the patients			Patients by type of cancer				
			Colorectal cancer (N=10,353)		Breast cancer (N=9546)		Lung cancer (N=12,671)
Age at index (years), mean (SD)			62 (13.2)		50 (11.4)		64 (12.7)
**Sex, n (%)**							
	Male	6116 (59.1)		62 (0.7)		9166 (72.3)	
	Female	4237 (40.9)		9484 (99.3)		3505 (27.7)	
**Number of patients who received chemotherapy by year range, n (%)**							
	1999–2002	265 (2.6)		646 (6.8)		524 (4.1)	
	2003–2006	516 (5.0)		829 (8.7)		617 (4.9)	
	2007–2010	672 (6.5)		965 (10.1)		738 (5.8)	
	2011–2014	852 (8.2)		1373 (14.4)		775 (6.1)	
	2015–2018	912 (8.8)		2111 (22.1)		1127 (8.9)	
Number of patients who underwent surgery, n (%)			3760 (36.3)		5541 (58.0)		1776 (14.0)
Baseline absolute neutrophil count/μL, mean (SD)			5582 (4403)		3750 (3199)		6517 (5283)
**Number of patients who received chemotherapy, n (%)**							
	First-line treatment	3151 (30.4)		5568 (58.3)		1593 (12.5)	
	Second-line treatment	1212 (11.7)		4739 (49.6)		888 (7.0)	
	Third-line treatment	506 (4.8)		4005 (41.9)		521 (4.1)	
	Fourth-line treatment	234 (2.2)		3573 (37.4)		336 (2.6)	

### Validation of the Accuracy of TRACER

Table 2 shows the values obtained for the accuracy of the algorithm-derived episode records compared to the clinical notes. The positive predictive value for the type of regimen was over 95% for the FULV regimen, and the chemotherapy type was estimated precisely for the entire episode for FOLFOX, FOLFIRI, and capecitabine monotherapy. The number of treatment cycles was correctly inferred in an average of 85.6% of episode records for validated regimens. The mean difference between the number of cycles in the episode records and the description in the clinical notes was less than one cycle.

**Table 2:** Validation of chemotherapy episodes compared to chart review.

Treatment regimen	Without information, n	Positive predictive value^a,b^ of regimen type, n/N (%)	Accuracy^c^ of treatment cycle number, %	Mean absolute error	Root mean square error
FULV^d^	30	67/70 (95)	94	0.1	0.4
FOLFOX^e^	8	92/92 (100)	87	0.3	0.6
FOLFIRI^f^	21	79/79 (100)	89	0.4	1.4
Capecitabine monotherapy	65	35/35 (100)	73	0.7	1.5

^a^For each regimen, 100 cases were randomly sampled and reviewed. The information for chemotherapy was not available in the discharge summary in 30, 8, 21, and 65 cases with FULV, FOLFOX, FOLFIRI, and capecitabine monotherapy, respectively.

^b^Positive predictive value for the matched cases for the type of regimen in the manual comparison of generated episode records with the contents of the clinical notes.

^c^The percentage of matched cases for the number of treatment cycles in the manual comparison of generated episode records with the contents of the clinical notes.

^d^FULV: fluorouracil and leucovorin.

^e^FOLFOX: fluorouracil, leucovorin, and oxaliplatin.

^f^FOLFIRI: fluorouracil, leucovorin, and irinotecan.

### Patterns of Chemotherapy Treatment

The trends in chemotherapy regimen used in the AUSOM database are shown in Multimedia Appendix 2. The number of chemotherapy regimens—including targeted anticancer agents (eg, osimertinib, ceritinib, and crizotinib) and immunotherapies (eg, nivolumab and pembrolizumab)—for lung cancer increased since 2016. On average, 31.2% of patients received the FOLFOX regimen throughout the years, followed by FULV, which was administered to 20.3% of patients. As of 2012, the proportion of patients receiving chemotherapy with targeted anticancer drugs (eg, bevacizumab or cetuximab) had increased gradually. Tamoxifen use also increased gradually among patients with breast cancer. Multimedia Appendix 3 shows the trends in chemotherapy regimen use in the KDH database. The most frequently used regimen for colorectal cancer in the KDH database was also FOLFOX (27.3% on average), followed by FULV, with an average of 23%. The use of regimens including targeted anticancer drugs for colorectal cancer in the KDH database increased since 2013. From 2013-2016, gefitinib monotherapy was the most frequently used regimen for lung cancer, which was similar to the trends of the AUSOM database. Among the patients with breast cancer in the KDH database, the use of trastuzumab increased sharply since 2014.

**Figure 4:**
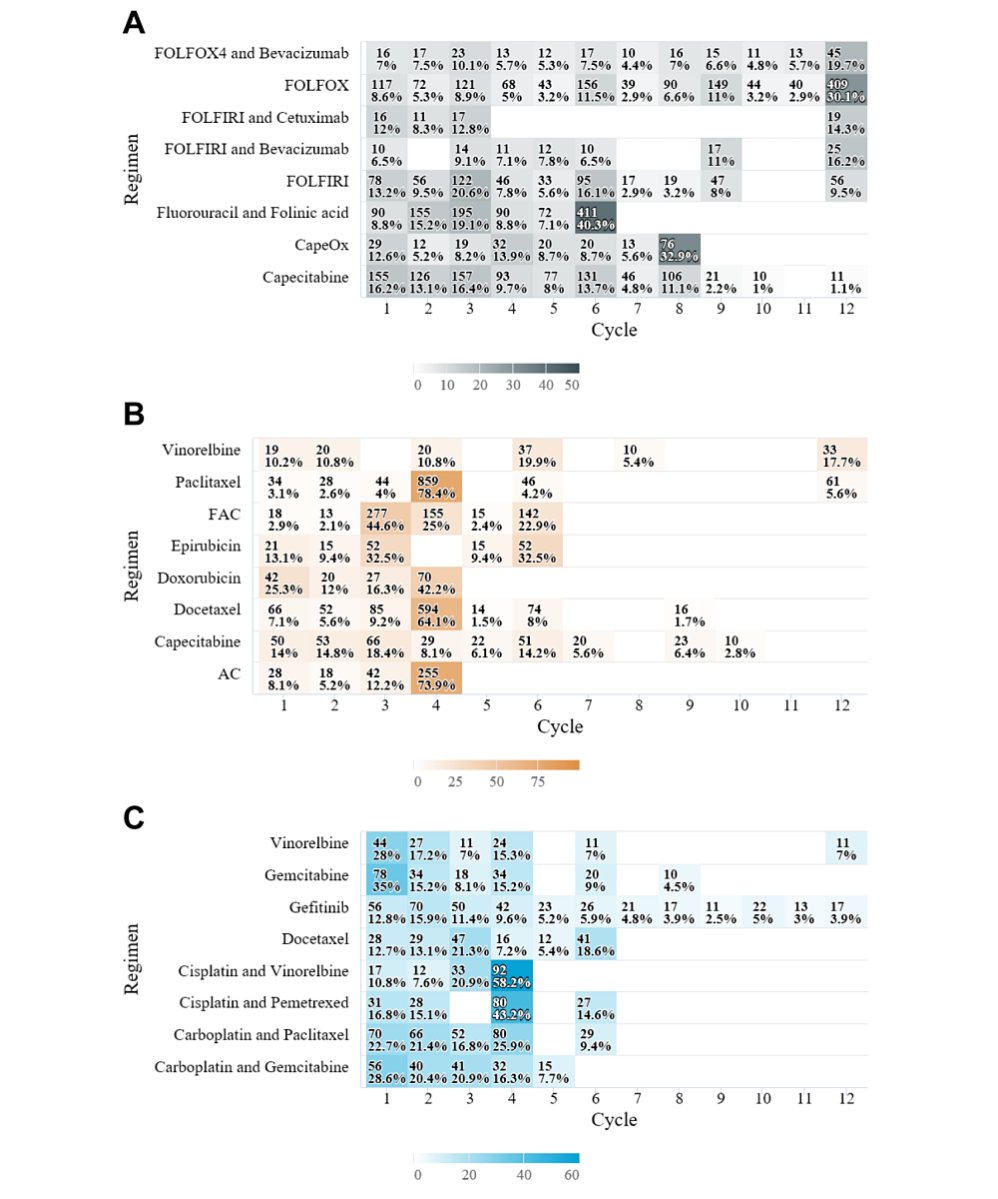
Heat map of patient distribution for cycle iteration by regimen type in the Ajou University School of Medicine database. The number of patients with (A) colorectal cancer, (B) breast cancer, and (C) lung cancer is shown; treatment iteration count is represented by color saturation, with a darker shade representing a higher number of patients. Cells with <10 patients are not reported. AC: doxorubicin and cyclophosphamide; CapeOx: capecitabine and oxaliplatin; FAC: fluorouracil, doxorubicin, and cyclophosphamide; FOLFIRI: fluorouracil, leucovorin, and irinotecan; FOLFOX: fluorouracil, leucovorin, and oxaliplatin.

The distribution of patients in both EHR databases over the number of iterated chemotherapy cycles is depicted as a heat map. In the heat map of the AUSOM database, the most prevalent number of repeated cycles in the FULV, FOLFOX, and capecitabine and oxaliplatin (CapeOx) regimens for colorectal cancer was consistent with the recommendations of the HemOnc regimen protocol for the adjuvant setting (6, 12, and 8 cycles, respectively; Figure 4). In addition, the most prevalent number of treatment iterations for paclitaxel monotherapy, doxorubicin monotherapy, and cyclophosphamide and doxorubicin (AC) targeting breast cancer was also consistent with the general recommendations (4 cycles in certain cases). “Cisplatin and vinorelbine” and “cisplatin and pemetrexed” for lung cancer were also consistent with the recommendations (4 cycles in certain cases). In the KDH database, the number of patients with colorectal cancer treated with 12 cycles made up the largest proportion among the patients who received the FOLFOX regimen (Multimedia Appendix 4). A total of four regimens—fluorouracil, epirubicin, and cyclophosphamide (FEC); fluorouracil, doxorubicin, and cyclophosphamide (FAC); cyclophosphamide, methotrexate, and fluorouracil (CMF); and Taxotere—for breast cancer were mostly repeated 6 times, and each of the regimen protocols in HemOnc included 6 cycles in certain cases.

### Trajectory of Cancer Treatment

**Figure 5:**
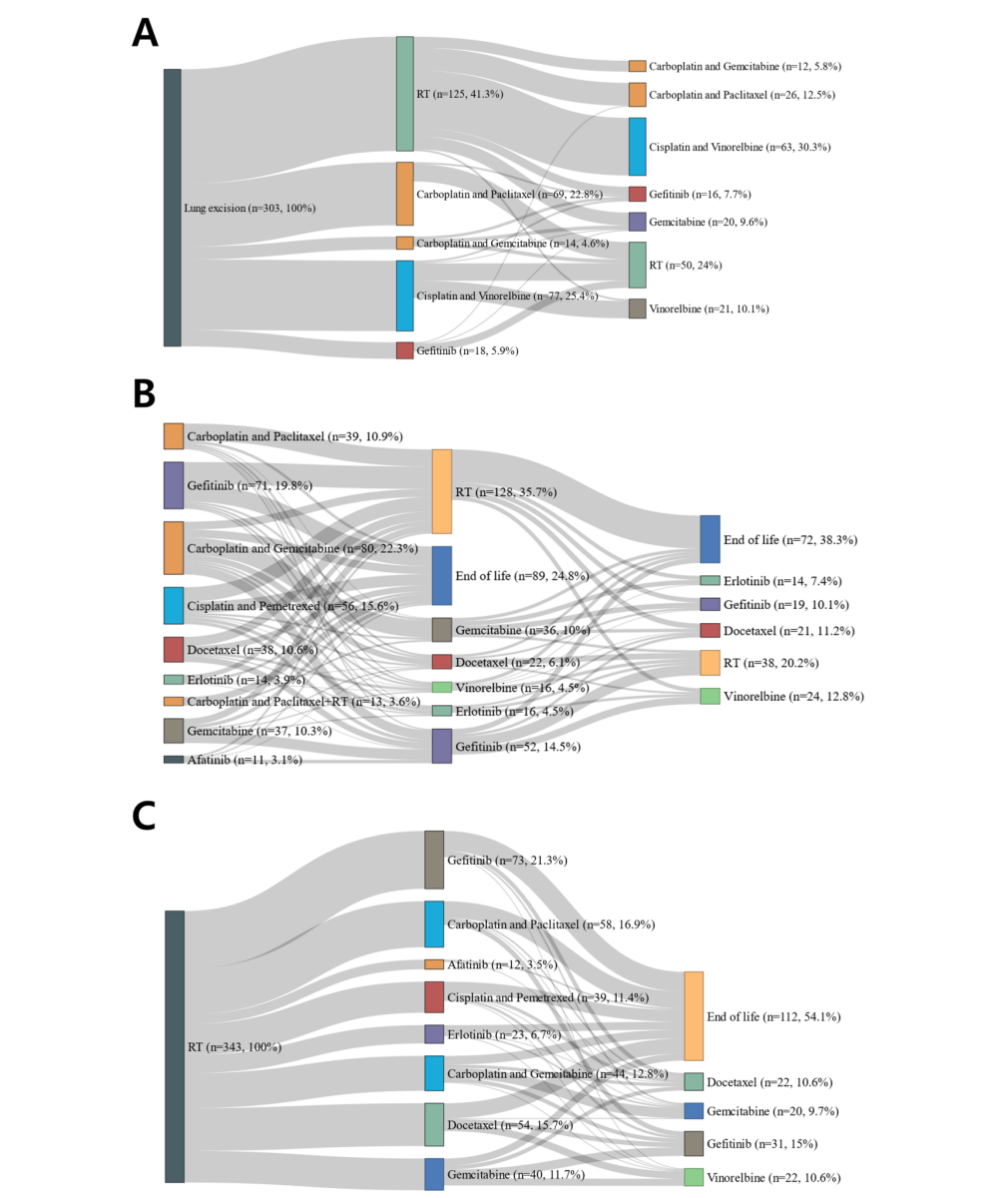
Anticancer treatment trajectories of patients with lung cancer in the Ajou University School of Medicine database. The treatment trajectories of patients with lung cancer were classified according to the type of first-line treatment: (A) surgery, (B) chemotherapy or chemotherapy with radiation, and (C) radiation therapy. The height of each node represents the population of patients in the corresponding treatment line or therapy. The number of patients who progressed to the next line of treatment is illustrated using gray lines. The chemotherapy regimen changes or the transition between types of treatment were regarded as a treatment line transition. The percentage on the label covers the proportion of the number of patients to all patients in the identical line of trajectory. As the large number of nodes hinders the purpose of the visualizations within a graphical summary, the nodes are truncated at the third node. For the same reason, the nodes for patient count under 10 were removed. AUSOM: Ajou University School of Medicine; RT: radiation therapy.

Figure 5 shows the treatment trajectories of patients with lung cancer in the AUSOM database. Among a total of 1120 patients, lung excision–radiation therapy–cisplatin and vinorelbine (n=63, 5.6%) was the most prevalent trajectory. The treatment trajectories of patients with colorectal or breast cancer were displayed, regardless of which first-line treatment was used (Multimedia Appendix 5). Among the treatment trajectories of patients with colorectal cancer or breast cancer that included at least three treatments, colectomy–FOLFOX–FOLFIRI (n=78, 4%) and mastectomy–AC–paclitaxel monotherapy (n=236, 5%) were the trajectories with the highest proportions, respectively. The 10 most frequent trajectories according to cancer type are described in Multimedia Appendix 6.

**Figure 6:**
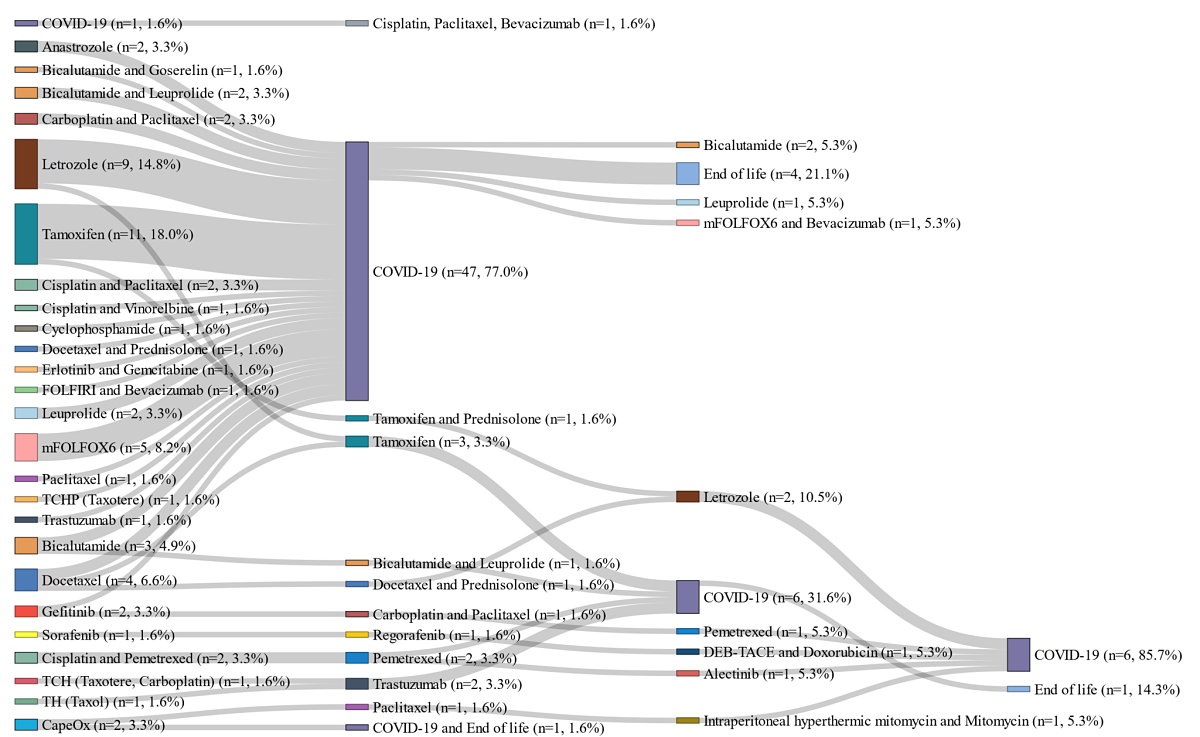
Anticancer treatment trajectories of patients with COVID-19. Sankey plot of the treatment trajectories of patients with COVID-19, including episodes of anticancer chemotherapy between 2017 and 2020. Each node represents the chemotherapy regimen used for cancer treatment. The percentage on the label covers the proportion of the patient number in each node in the same trajectory phase. As the large number of nodes hinders the purpose of the visualizations within a graphical summary, the nodes are truncated at the fourth node. For the same reason, the nodes for patient count <5 were removed.

The treatment trajectories of patients in the KDH database are illustrated in Multimedia Appendix 7. Among the patients with colorectal cancer, FOLFOX was the most frequently used first-line (n=52, 17.9%) and second-line (n=84, 28.9%) regimen in the trajectory. For breast cancer, Taxotere was the most frequent first-line chemotherapy before mastectomy (n=58, 17.3%). Gefitinib was the most widely used first-line regimen among patients with lung cancer (n=30, 10.5%). Figure 6 shows the treatment trajectories of patients with malignant neoplasm who also had COVID-19. Of the 7590 patients nationwide with a diagnosis of COVID-19, we identified 382 patients with a history of cancer. Among them, a total of 62 patients had an episode of chemotherapy. Most of the patients received only one line of treatment between 2017-2020, before COVID-19 infection (n=47). The trajectory included 6 patients with a node of end of life.

### Timing of Chemotherapy-Induced Neutropenia

**Figure 7:**
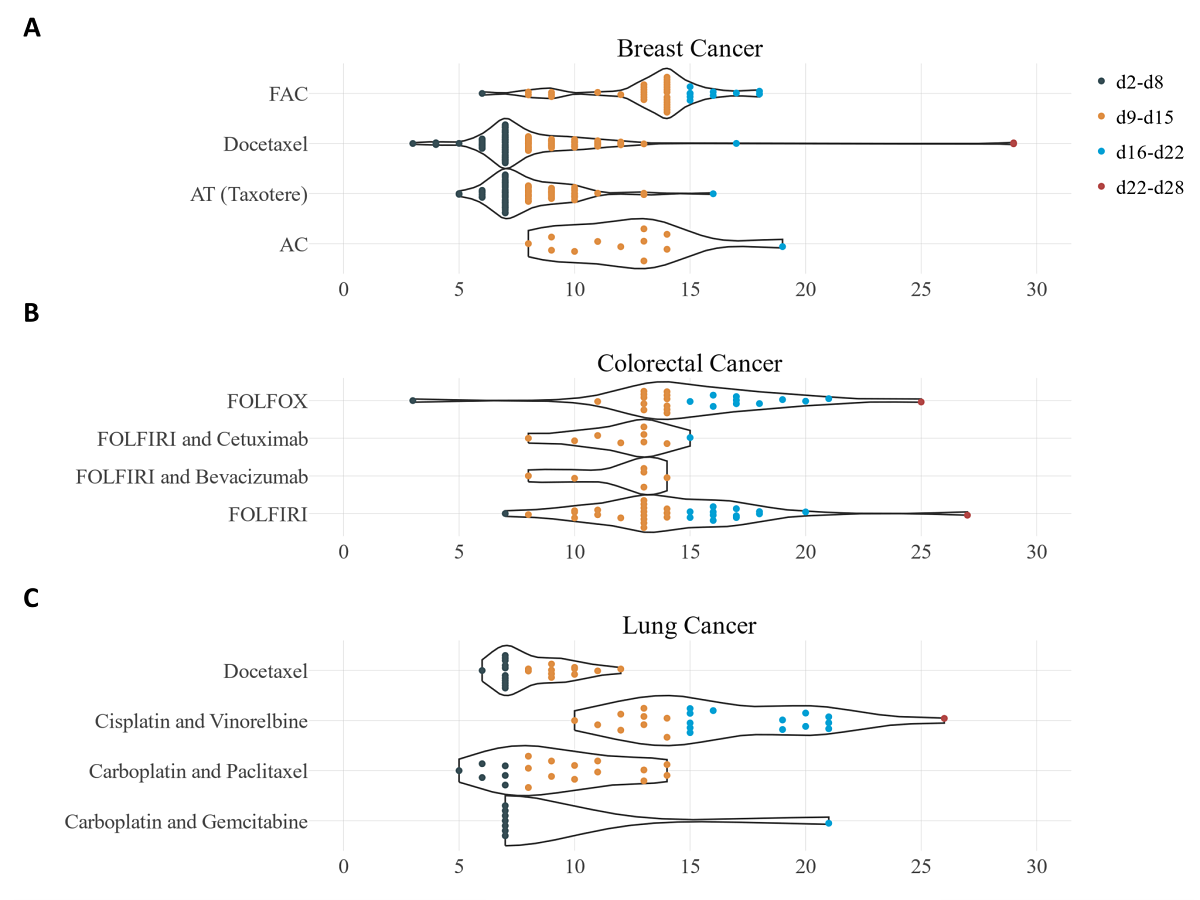
Trends in neutropenia onset time according to regimen. Time of onset of chemotherapy-induced (febrile) neutropenia event after the first exposure to chemotherapy among patients with (A) breast cancer, (B) colorectal cancer, and (C) lung cancer at the Ajou University School of Medicine. Each dot represents the neutropenia event of a distinct patient. The events are categorized in a 7-day range. The violin plot represents the trends in the frequency on each day from chemotherapy exposure. AC: doxorubicin and cyclophosphamide; FAC: fluorouracil, doxorubicin, and cyclophosphamide; FOLFIRI: fluorouracil, leucovorin, and irinotecan; FOLFOX: fluorouracil, leucovorin, and oxaliplatin.

Figure 7 shows the time of onset of the CIN/FN event for each patient in the AUSOM database. The episodes of neutropenia among patients with colorectal cancer were clustered between days 9 and 15. Compared with the regimens used for colorectal cancer, the neutropenia events that were recorded after docetaxel monotherapy and Taxotere treatment for breast cancer and after carboplatin and gemcitabine for lung cancer generally began one week earlier (days 2-8). We illustrated the incidence of neutropenia events in each cycle of treatment in Multimedia Appendix 8. Regardless of the cancer type, the incidence of CIN/FN events was high during the first cycle, with the exception of the FOLFOX regimen for colorectal cancer and the carboplatin and paclitaxel regimen for lung cancer. Finally, neutropenia occurred more frequently during the Taxotere regimen for breast cancer (75.3%), which includes doxorubicin and docetaxel as constituent drugs.

## Discussion

### Overview

This study described a system for analyzing the treatment patterns and trajectories of patients with cancer based on the oncology extension model in the OMOP-CDM. The proposed algorithm (TRACER) for extracting chemotherapy episodes at the regimen level effectively generated the treatment episodes for patients with cancer. This approach illustrates how laborious manual curation can be replaced with an automatic extraction system. The obtained episodes were validated by reviewing clinical notes, which revealed that the type of regimen or the number of treatment cycles were estimated with high accuracy. We also demonstrated the usefulness of the proposed system by performing a pilot study investigating the onset time of CIN/FN across various chemotherapy regimens.

### Principal Findings

Comprehensive clinical information, including longitudinal treatment sequences and the various clinical outcomes of patients with cancer, is not available in nationwide cancer registries, such as the Surveillance, Epidemiology, and End Results Program [16-18] or the Korea Central Cancer Registry [19,20]. Large-scale real-world data derived from EHRs [21] and administrative claims data [17] of a standardized data network can support the timely assessment of the characterization and quality of routine clinical practice and active pharmacovigilance across institutions or countries.

The unexpectedly rapid spread of COVID-19 revealed an urgent unmet need for the timely retrieval of detailed data for patients with cancer, to provide relevant evidence for the management of patients with cancer during the pandemic period [22]. Although conventional cancer registries have failed to provide these data to researchers, the secondary use of EHRs and claims databases can promptly provide valuable insights into the impact of a novel infection on patients with cancer [13]. The TRACER was able to generate records to describe the trajectory of cancer treatment and death of patients with COVID-19, which may be helpful for identifying the relationship between cancer treatment and a fatal case of COVID-19.

We demonstrated how electronically captured data elements can support clinical research using longitudinal detailed clinical data. FN is one of the most common oncologic emergencies [23] and is associated with considerable morbidity and mortality [24]. Although it is well known that the risk of CIN/FN is highest during the first cycle of chemotherapy for solid tumors or lymphoma [25,26], the exact time of occurrence of CIN/FN in various regimens is largely unknown. We found that CIN/FN events were more frequent in the first cycle of chemotherapy, and that regimens that included docetaxel or doxorubicin were followed by a greater number of CIN/FN events, which is compatible with reported findings [27]. CIN/FN events usually occurred relatively early (days 2-8) in patients who received regimens including docetaxel or carboplatin compared with those treated with other regimens (days 7-13).

### Limitations

This study had several limitations. First, only four of the regimens were validated through manual review; thus, it was not clear whether episodes for the other types of regimen can also be precisely estimated. Nevertheless, the patterns of treatment cycle repetition showed that the extracted records were concordant with the standard protocols of the regimens, suggesting that the algorithm could correctly interpret the variable regimens. A second limitation was that the relatively low rate of ascertained treatment episodes (maximum of 58% among patients with breast cancer in the AUSOM database) suggests that treatment episodes may have been missed, although many patients with cancer are treated with surgery or radiation alone and would have appropriately not been captured. This may be due to the fact that the algorithm extracts only the regimens included in the HemOnc vocabulary. The flexible structure of HDAC, which allows the addition of user-defined rules for specific regimens, has the potential to mitigate the missing rate of treatment episodes for a particular study using a fine-tuned algorithm.

### Conclusions

We developed a technique to generate episodes for chemotherapies included in the oncology module of the OMOP-CDM and to analyze treatment patterns in patients with cancer. We demonstrated that the proposed process is reproducible and scalable across a distributed data network. Our findings suggest that a generalizable strategy of characterizing treatment trajectories from harmonized observational databases can promptly determine the characteristics of clinical events, thus enabling the generation of real-world evidence for abrupt pandemic crises. Further research is required to generate statistical evidence for clinical outcomes between regimen types.

## Appendix

Multimedia Appendix 1 Chemotherapy episodes from the Ajou University School of Medicine database. The 10 most frequently used chemotherapy regimens for colorectal cancer, breast cancer, and lung cancer are listed.

Multimedia Appendix 2 Trends of chemotherapy regimen use within the Ajou University School of Medicine database. The proportions of chemotherapy regimen uses for patients with (A) colorectal cancer, (B) breast cancer, and (C) lung cancer by year from 2008-2018 in the Ajou University School of Medicine database are shown.

Multimedia Appendix 3 Trends of chemotherapy regimen use within the Kangdong Sacred Heart Hospital database. The proportions of chemotherapy regimen uses for patients with (A) colorectal cancer, (B) breast cancer, and (C) lung cancer by year from 2008-2018 in the Kangdong Sacred Heart Hospital database are shown.

Multimedia Appendix 4 Heat map of patient distribution for cycle iteration by regimen type in the Kangdong Sacred Heart Hospital database. The number of patients with (A) colorectal cancer, (B) breast cancer, and (C) lung cancer is shown; treatment iteration counts are represented by a color saturation difference.

Multimedia Appendix 5 Trends of chemotherapy regimen use from the Kangdong Sacred Heart Hospital database. The proportions of chemotherapy regimen uses for patients with (A) colorectal cancer, (B) breast cancer, and (C) lung cancer by year from 2008-2018 in the Kangdong Sacred Heart Hospital database are shown.

Multimedia Appendix 6 The list of treatment trajectories of patients with cancer from the Ajou University School of Medicine database.

Multimedia Appendix 7 Anticancer treatment trajectories for patients with cancer in the Kangdong Sacred Heart Hospital database. The treatment trajectories of patients with (A) colorectal cancer, (B) breast cancer, and (C) lung cancer in the Kangdong Sacred Heart Hospital database are shown.

Multimedia Appendix 8 Incidence of neutropenia by treatment cycle. The histogram of incidence of the first neutropenia event by cycle and regimen for (A) colorectal cancer, (B) lung cancer, and (C) breast cancer.

## Abbreviations

AC: cyclophosphamide and doxorubicin
ANC: absolute neutrophil count
AUSOM: Ajou University School of Medicine
CapeOx: capecitabine and oxaliplatin
CDM: common data model
CIN/FN: chemotherapy-induced (febrile) neutropenia
CMF: cyclophosphamide, methotrexate, and fluorouracil
CRCAE: Common Terminology Criteria for Adverse Events
EHR: electronic health record
FAC: fluorouracil, doxorubicin, and cyclophosphamide
FEC: fluorouracil, epirubicin, and cyclophosphamide
FOLFIRI: fluorouracil, leucovorin, and irinotecan
FOLFOX: fluorouracil, leucovorin, and oxaliplatin
FULV: fluorouracil and folinic acid
HDAC: Hierarchical Description for Administration of Chemotherapy
HIRA: Health Insurance Review and Assessment Service
JSON: JavaScript Object Notation
KDH: Kangdong Sacred Heart Hospital
OHDSI: Observational Health Data Sciences and Informatics
OMOP: Observational Medical Outcomes Partnership
TRACER: Tool for Regimen-level Abstraction of Chemotherapy Episode Records
