# Supplementary material for: Characterizing the Anticancer Treatment Trajectory and Pattern in Patients Receiving Chemotherapy for Cancer Using Harmonized Observational Databases: Retrospective Study
Source: JMIR Med Inform. 2021 Apr 6;9(4):e25035. doi: 10.2196/25035 (PMC8058693; doi:10.2196/25035)
Supplement: Multimedia Appendix 1 [file medinform_v9i4e25035_app1.docx]

Multimedia Appendix 1. Chemotherapy episodes of the Ajou University School of Medicine database.^a-l^

| Colorectal Cancer | |
| --- | --- |
| Type of the regimen | N |
| Fluorouracil and Folinic acid | 2,472 |
| FOLFOX^a^ | 1,357 |
| Capecitabine monotherapy | 997 |
| FOLFIRI^b^ | 593 |
| FOLFOX4 and Bevacizumab | 228 |
| CapeOx | 228 |
| FOLFIRI and Bevacizumab | 154 |
| FOLFIRI and Cetuximab | 132 |
| Cetuximab monotherapy | 40 |
| Regorafenib monotherapy | 33 |
| FOLFOX4 and Cetuximab | 28 |
| CapeOx and Bevacizumab | 3 |
| Breast Cancer | |
| Type of the regimen | N |
| Tamoxifen monotherapy | 2,624 |
| Letrozole monotherapy | 1,679 |
| Paclitaxel monotherapy | 1,104 |
| Docetaxel monotherapy | 938 |
| Toremifene monotherapy | 707 |
| FAC | 621 |
| Trastuzumab monotherapy | 561 |
| Capecitabine monotherapy | 369 |
| AC | 345 |
| Anastrozole monotherapy | 275 |
| Vinorelbine monotherapy | 202 |
| Doxorubicin monotherapy | 172 |
| Epirubicin monotherapy | 162 |
| Exemestane monotherapy | 142 |
| TH (Taxol) | 113 |
| AT (Taxotere) | 108 |
| FEC | 90 |
| Eribulin monotherapy | 87 |
| CMF | 84 |
| TH (Taxotere) | 81 |
| Everolimus and Exemestane | 76 |
| Capecitabine and Lapatinib | 67 |
| AT (Taxol) | 29 |
| THP (Taxotere) | 24 |
| Lung Cancer | |
| Type of the regimen | N |
| Gemcitabine monotherapy | 456 |
| Gefitinib monotherapy | 439 |
| Carboplatin and Paclitaxel | 302 |
| Vinorelbine monotherapy | 269 |
| Docetaxel monotherapy | 222 |
| Carboplatin and Gemcitabine | 198 |
| Cisplatin and Pemetrexed | 184 |
| Cisplatin and Vinorelbine | 158 |
| Erlotinib monotherapy | 128 |
| Cisplatin and Docetaxel | 124 |
| Afatinib monotherapy | 80 |
| Cisplatin and Paclitaxel | 73 |
| Gemcitabine and Vinorelbine | 64 |
| Nivolumab monotherapy | 36 |
| Cisplatin and Gemcitabine | 26 |
| Pembrolizumab monotherapy | 22 |
| Osimertinib monotherapy | 21 |
| Crizotinib monotherapy | 12 |
| Ceritinib monotherapy | 3 |

^a^FOLFOX: fluorouracil, leucovorin and oxaliplatin.

^b^FOLFIRI: fluorouracil, leucovorin and irinotecan.

^c^CapeOX: capecitabine and oxaliplatin.

^d^FAC: fluorouracil, doxorubicin, and cyclophosphamide.

^e^AC: doxorubicin and cyclophosphamide.

^f^TH (Taxol): taxol and trastuzumab.

^g^AT (Taxotere): doxorubicin and docetaxel.

^h^FEC: fluorouracil, epirubicin, and cyclophosphamide.

^i^CMF: cyclophosphamide, methotrexate, and fluorouracil

^j^TH (Taxotere): docetaxel and trastuzumab

^k^AT (Taxol): doxorubicin and paclitaxel

^l^THP (Taxotere): docetaxel, trastuzumab, and pertuzumab
