# Supplementary material for: Characterizing the Anticancer Treatment Trajectory and Pattern in Patients Receiving Chemotherapy for Cancer Using Harmonized Observational Databases: Retrospective Study
Source: JMIR Med Inform. 2021 Apr 6;9(4):e25035. doi: 10.2196/25035 (PMC8058693; doi:10.2196/25035)

Multimedia Appendix 3. Trends of chemotherapy regimen use of the Kangdong Sacred Heart Hospital database. The proportion share of chemotherapy regimen uses for patients with (a) colorectal cancer, (b) breast cancer, and (c) lung cancer by year from 2008 to 2018 in Kangdong Sacred Heart Hospital database.


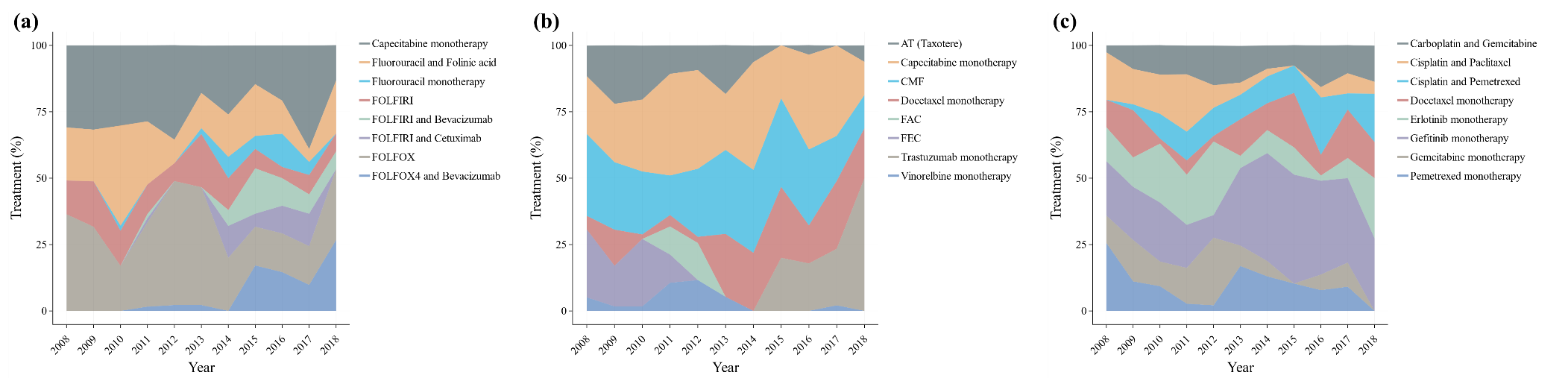

Supplement: Multimedia Appendix 3 [file medinform_v9i4e25035_app3.docx]
