# Supplementary material for: Characterizing the Anticancer Treatment Trajectory and Pattern in Patients Receiving Chemotherapy for Cancer Using Harmonized Observational Databases: Retrospective Study
Source: JMIR Med Inform. 2021 Apr 6;9(4):e25035. doi: 10.2196/25035 (PMC8058693; doi:10.2196/25035)

Multimedia Appendix 4. Heatmap of patient distribution for cycle iteration by regimen types in the Kangdong Sacred Heart Hospital database. The number of patients with cancer: (a) colorectal cancer, (b) breast cancer, and (c) lung cancer; for treatment iteration counts is represented by color saturation difference.


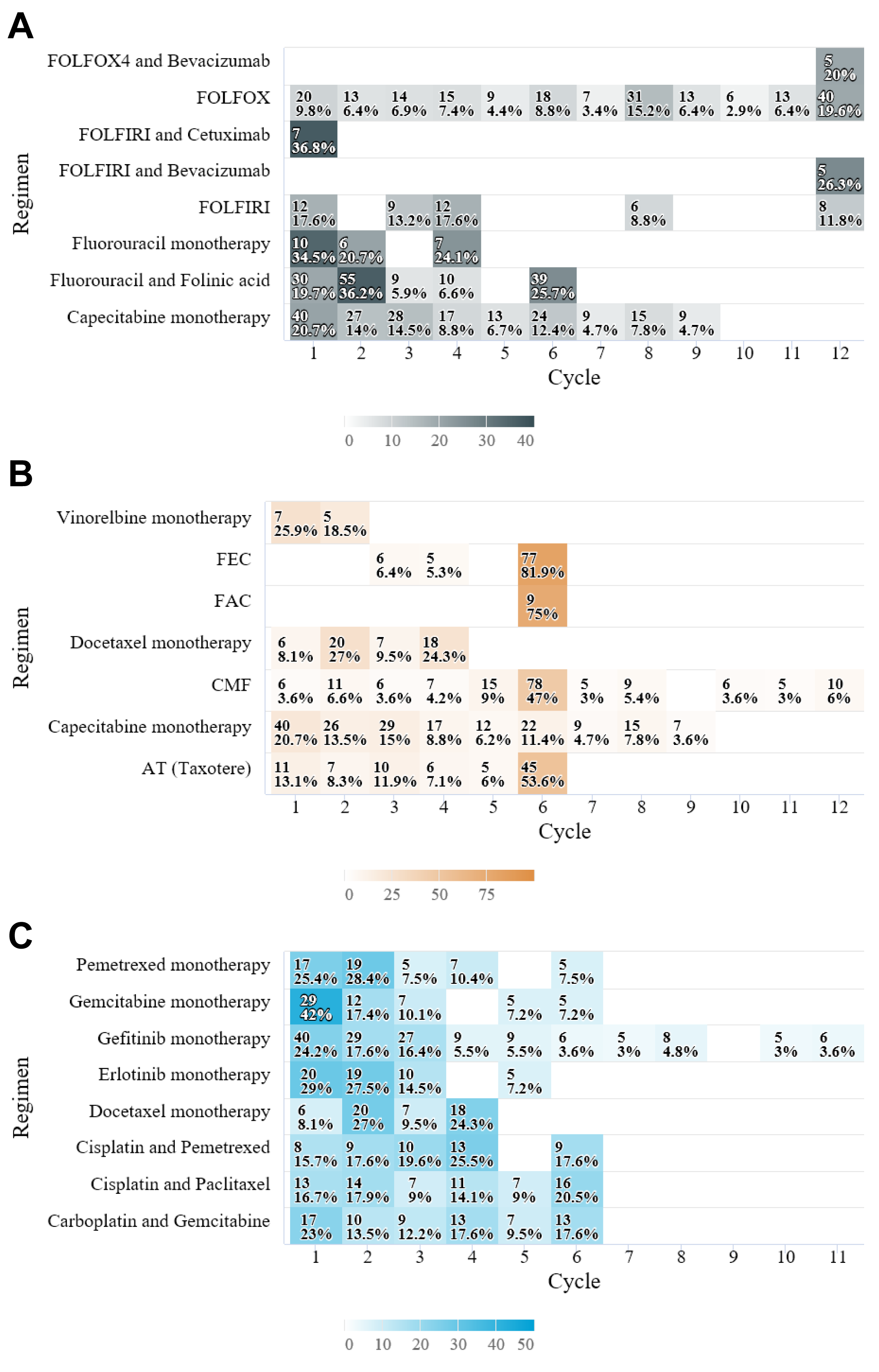

Supplement: Multimedia Appendix 4 [file medinform_v9i4e25035_app4.docx]
