# Supplementary material for: Characterizing the Anticancer Treatment Trajectory and Pattern in Patients Receiving Chemotherapy for Cancer Using Harmonized Observational Databases: Retrospective Study
Source: JMIR Med Inform. 2021 Apr 6;9(4):e25035. doi: 10.2196/25035 (PMC8058693; doi:10.2196/25035)

Multimedia Appendix 5. Anticancer treatment trajectories for patient with colorectal or breast cancer in the Ajou University School of Medicine database. The treatment trajectories of patients with (a) colorectal cancer and (b) breast cancer in AUSOM database were shown. AUSOM: Ajou University School Of Medicine; RT: Radiation Therapy


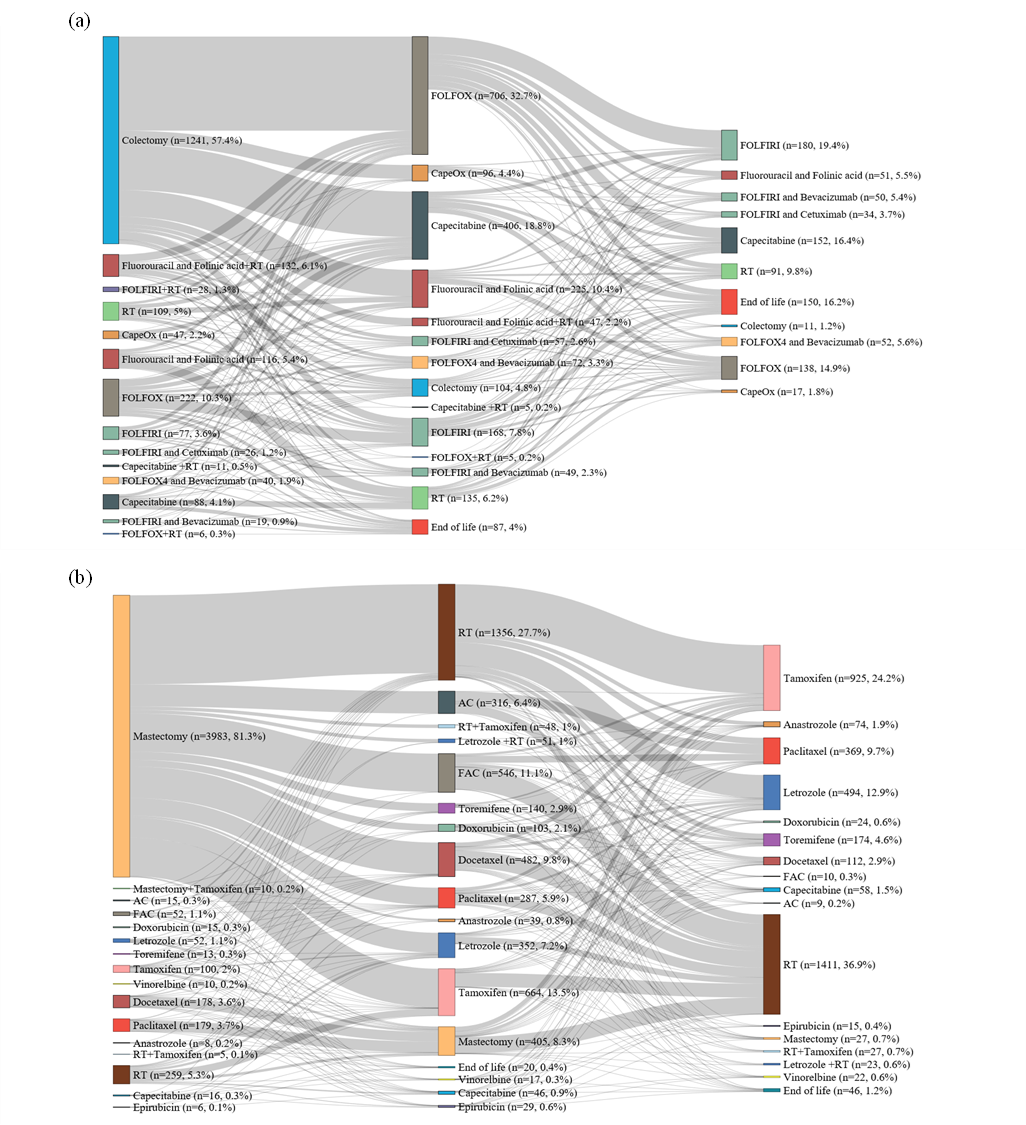

Supplement: Multimedia Appendix 5 [file medinform_v9i4e25035_app5.docx]
