# Supplementary material for: Characterizing the Anticancer Treatment Trajectory and Pattern in Patients Receiving Chemotherapy for Cancer Using Harmonized Observational Databases: Retrospective Study
Source: JMIR Med Inform. 2021 Apr 6;9(4):e25035. doi: 10.2196/25035 (PMC8058693; doi:10.2196/25035)
Supplement: Multimedia Appendix 6 [file medinform_v9i4e25035_app6.docx]

Multimedia Appendix 6. The list of treatment trajectories of patients with cancer of the Ajou university school of Medicine database.

| **Colorectal Cancer** | | **Breast Cancer** | | **Lung Cancer** | |
| --- | --- | --- | --- | --- | --- |
| **Trajectory** | **(N = 1,935)** | **Trajectory** | **(N = 4,727)** | **Trajectory** | **(N = 1,120)** |
| Colectomy-FOLFOX-NA | 381 | Mastectomy-Tamoxifen monotherapy-NA | 1134 | Lung excision-RT-Cisplatin and Vinorelbine | 63 (5.62%) |
| Colectomy-Capecitabine monotherapy-NA | 242 | Mastectomy-Letrozole monotherapy-NA | 563 | Lung excision-Carboplatin and Paclitaxel-NA | 48 (4.29%) |
| Colectomy-Fluorouracil and Folinic acid-NA | 147 | Mastectomy-AC-Paclitaxel monotherapy | 236 | RT-Gefitinib -NA | 40 (3.57%) |
| Colectomy-FOLFOX-FOLFIRI | 78 | Mastectomy-Docetaxel monotherapy-Tamoxifen monotherapy | 186 | Lung excision-Cisplatin and Vinorelbine-NA | 36 (3.21%) |
| Colectomy-CapeOx-NA | 49 | Mastectomy-Docetaxel monotherapy-NA | 162 | RT-Carboplatin and Paclitaxel-NA | 28 (2.5%) |
| Fluorouracil and Folinic acid-FOLFOX-NA | 44 | Mastectomy-FAC-Paclitaxel monotherapy | 153 | Gefitinib -End of life-NA | 26 (2.32%) |
| FOLFOX-FOLFIRI-NA | 42 | Mastectomy-FAC-Toremifene monotherapy | 132 | Lung excision-RT-Carboplatin and Paclitaxel | 25 (2.23%) |
| Colectomy-FOLFOX-Capecitabine monotherapy | 36 | Mastectomy-Paclitaxel monotherapy-Tamoxifen monotherapy | 126 | RT-Gefitinib -End of life | 25 (2.23%) |
| Colectomy-Fluorouracil and Folinic acid-FOLFOX | 33 | Mastectomy-FAC-NA | 124 | RT-Carboplatin and Paclitaxel-End of life | 21 (1.88%) |
| Fluorouracil and Folinic acid-Colectomy-NA | 33 | Mastectomy-Toremifene monotherapy-NA | 123 | Lung excision-Carboplatin and Paclitaxel-RT | 19 (1.7%) |
