# Supplementary material for: Characterizing the Anticancer Treatment Trajectory and Pattern in Patients Receiving Chemotherapy for Cancer Using Harmonized Observational Databases: Retrospective Study
Source: JMIR Med Inform. 2021 Apr 6;9(4):e25035. doi: 10.2196/25035 (PMC8058693; doi:10.2196/25035)

Multimedia Appendix 7. Anticancer treatment trajectories for patient with cancer in Kangdong sacred heart hospital database. The treatment trajectories of patients with (a) colorectal cancer, (b) breast cancer, and (c) lung cancer at the Kangdong sacred heart hospital database.


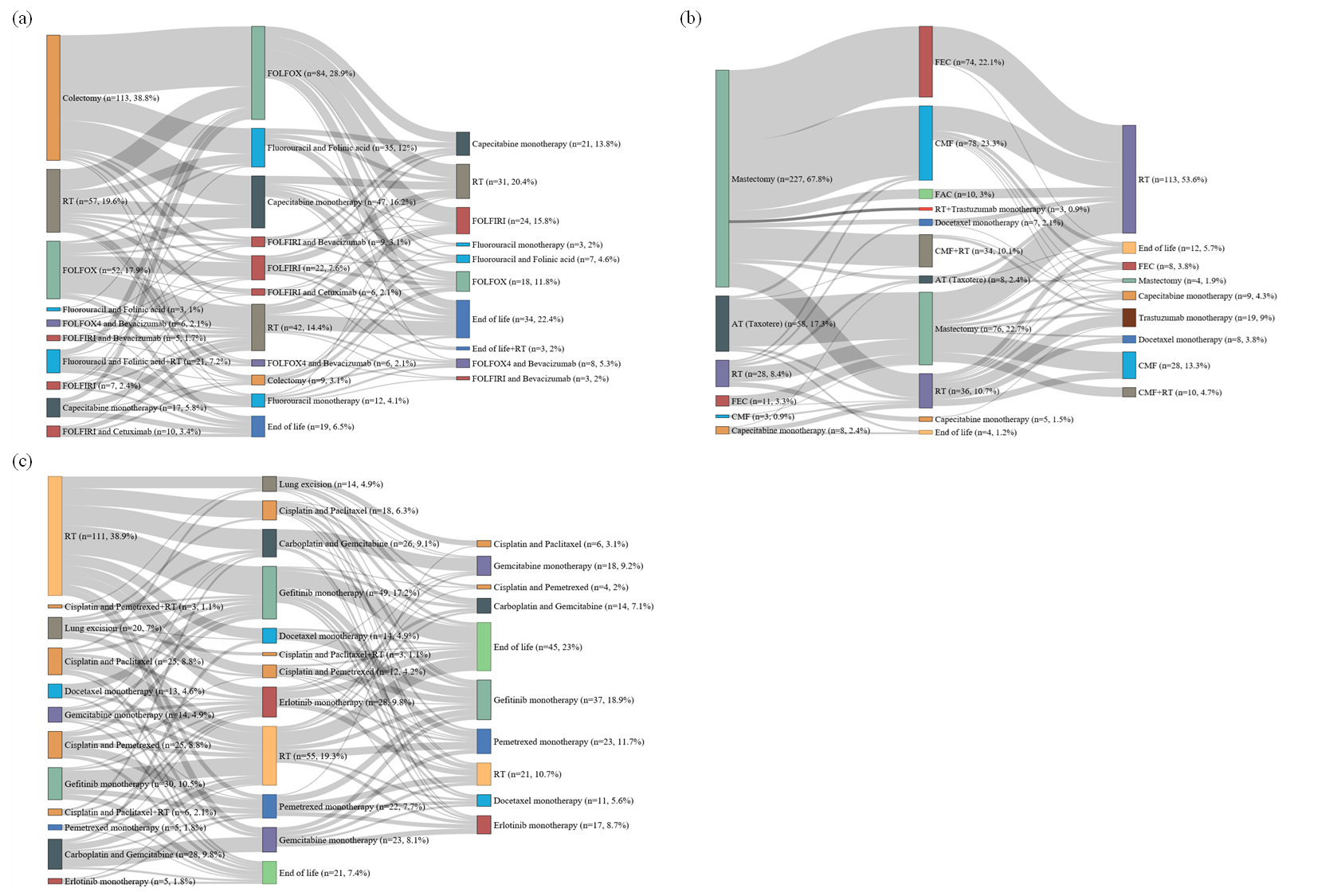

Supplement: Multimedia Appendix 7 [file medinform_v9i4e25035_app7.docx]
