# Supplementary material for: Characterizing the Anticancer Treatment Trajectory and Pattern in Patients Receiving Chemotherapy for Cancer Using Harmonized Observational Databases: Retrospective Study
Source: JMIR Med Inform. 2021 Apr 6;9(4):e25035. doi: 10.2196/25035 (PMC8058693; doi:10.2196/25035)

Multimedia Appendix 8. Incidence of neutropenia by treatment cycle. The histogram of incidence for first neutropenia event by cycle according to the regimen of (a) colorectal cancer, (b) lung cancer, and (c) breast cancer.


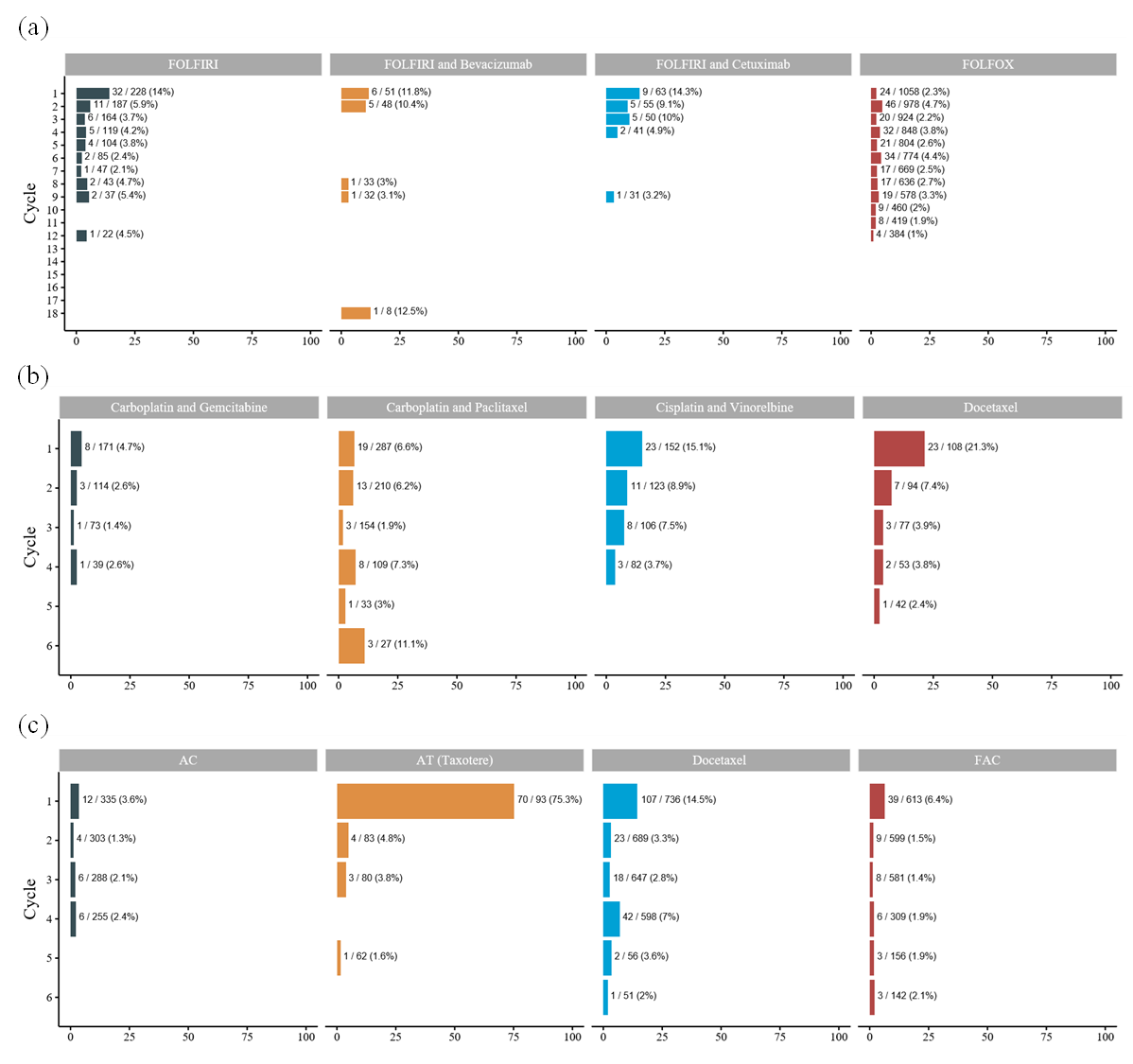

Supplement: Multimedia Appendix 8 [file medinform_v9i4e25035_app8.docx]
